# Supplementary material for: CD8+ T Cells Mediate Lethal Lung Pathology in the Absence of PD-L1 and Type I Interferon Signalling following LCMV Infection
Source: Viruses. 2024 Mar 1;16(3):390. doi: 10.3390/v16030390 (PMC10975266; doi:10.3390/v16030390)
Supplement: Supplementary file 1 [file viruses-16-00390-s001.zip › viruses-2887994-supplementary.pdf]

Supplementary Figures

Figure S1 Lethal pathology in DKO mice cannot be explained by plasma cytokine levels. Expression of cytokines and chemokines from the LEGENDplex™ Mouse Anti-Virus Response Panel in plasma isolated from WT, IFNAR1 KO, PD-L1 KO and IFNAR1 x PD-L1 DKO mice at day 8 post LCMV infection. Data is presented as mean ± SEM from a one of two independent experiments with consistent results using at least five mice per group and experiment.

Figure S2 Treating IFNAR KO mice with anti-PD-L1 does not reinvigorate the CD8$^+$ T cell response. (A, B) Percent weight lost and clinical score of IFNAR1 KO treated with an isotype control or anti-PD-L1 i.p. three times weekly for two weeks beginning at day 15 **(A)** or 24 **(B)** p.i. (1x10^3 PFU; day 38 p.i.). **(C, D)** Bar plots showing the frequency of CD8$^+$ T cells expressing granzyme B, IFN-γ and/or TNF after stimulation with LCMV $GP_{33\text{-}41}$ **(C)** or $NP_{396\text{-}404}$ **(D).** T cells were isolated from the spleen of mice infected intraperitoneally with LCMV-Arm (2x10^5 PFU; day 38 p.i.). **(E)** LCMV-NP RNA levels in arbitrary units (a.u.), detected in the liver and CNS from mice infected with LCMV-Arm (1x10^3 PFU; day 38 p.i.) as determined by an RNase protection assay. Representative results from two independent experiments are shown.

Figure S3 Gating strategy used to identify cell subsets in the lung of LCMV-infected WT, PD-L1 KO, IFNAR1 KO, IFNAR1 x PD-L1 DKO mice. Representative plots are shown for DKO uninfected and LCMV-infected mice. As anti-Ly6G mAb treatment masks the fluorescent detection of Ly6G, neutrophils were gated as Ly6C^{int}, CD48$^-$, CD11b$^+$. This comprises 98-99% of Ly6G$^+$ cells.

Figure S4 Percent of neutrophils and CD8$^+$ T cells depleted in the blood of anti-CD8 or anti-GR1 and anti-Ly6G treated IFNAR1 x PD-L1 DKO mice at day 7 p.i.. (A) Percent of neutrophils out of total leukocytes in the blood of DKO mice infected with 1x10^3 PFU LCMV and treated with anti-Ly6G

at day 7 p.i. (**B, C**) Number of neutrophils **(B)** and $CD8^+$ T cells **(C)**, their frequency out of total leukocytes and percent depleted in LCMV-infected DKO mice at day 8 p.i. that were untreated or treated with one or two isotype control mAbs, an anti-CD8 mAb or with anti-GR1 and anti-Ly6G mAbs. Numbers of neutrophils **(B)** and $CD8^+$ T cells **(C)** is shown with undepleted and isotype controls separate or aggregated as a single control group. Data is presented as mean ± SEM with 3-5 mice per group.

Figure S5 Expression of select genes in the lung and liver tissue of neutrophil- and $CD8^+$ T cell-depleted DKO LCMV-infected mice at day 7 p.i. Gene expression values were normalized to *Rpl13a*. Data is presented as mean ± SEM from a one experiment at least five mice per group and experiment.

Figure S1

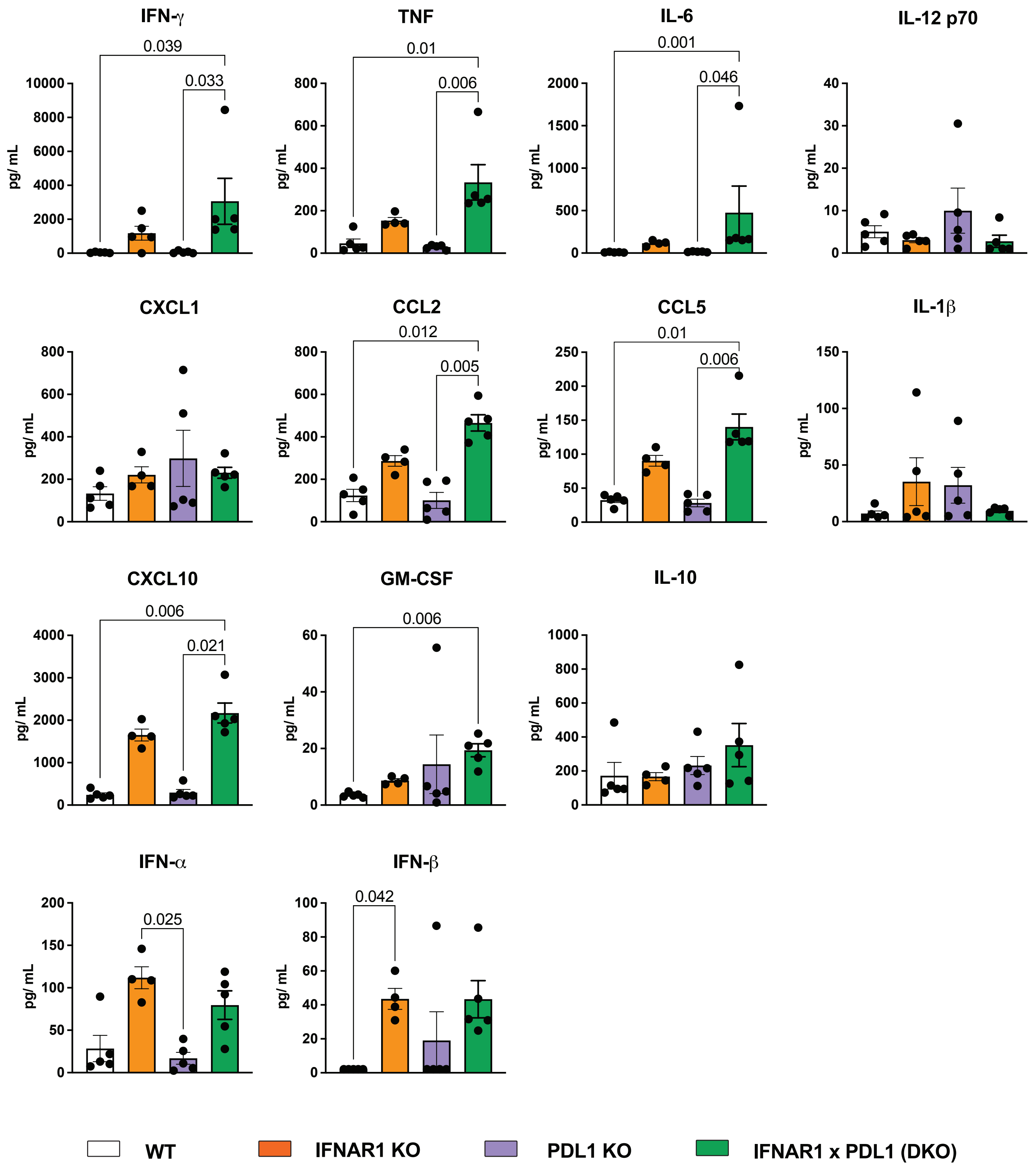

IFN-γ
0.039
0.033
TNF
0.01
0.006
IL-6
0.001
0.046
IL-12 p70
CXCL1
CCL2
0.012
0.005
CCL5
0.01
0.006
IL-1β
CXCL10
0.006
0.021
GM-CSF
0.006
IL-10
IFN-α
0.025
IFN-β
0.042
pg/ mL
WT
IFNAR1 KO
PDL1 KO
IFNAR1 x PDL1 (DKO)

Figure S2

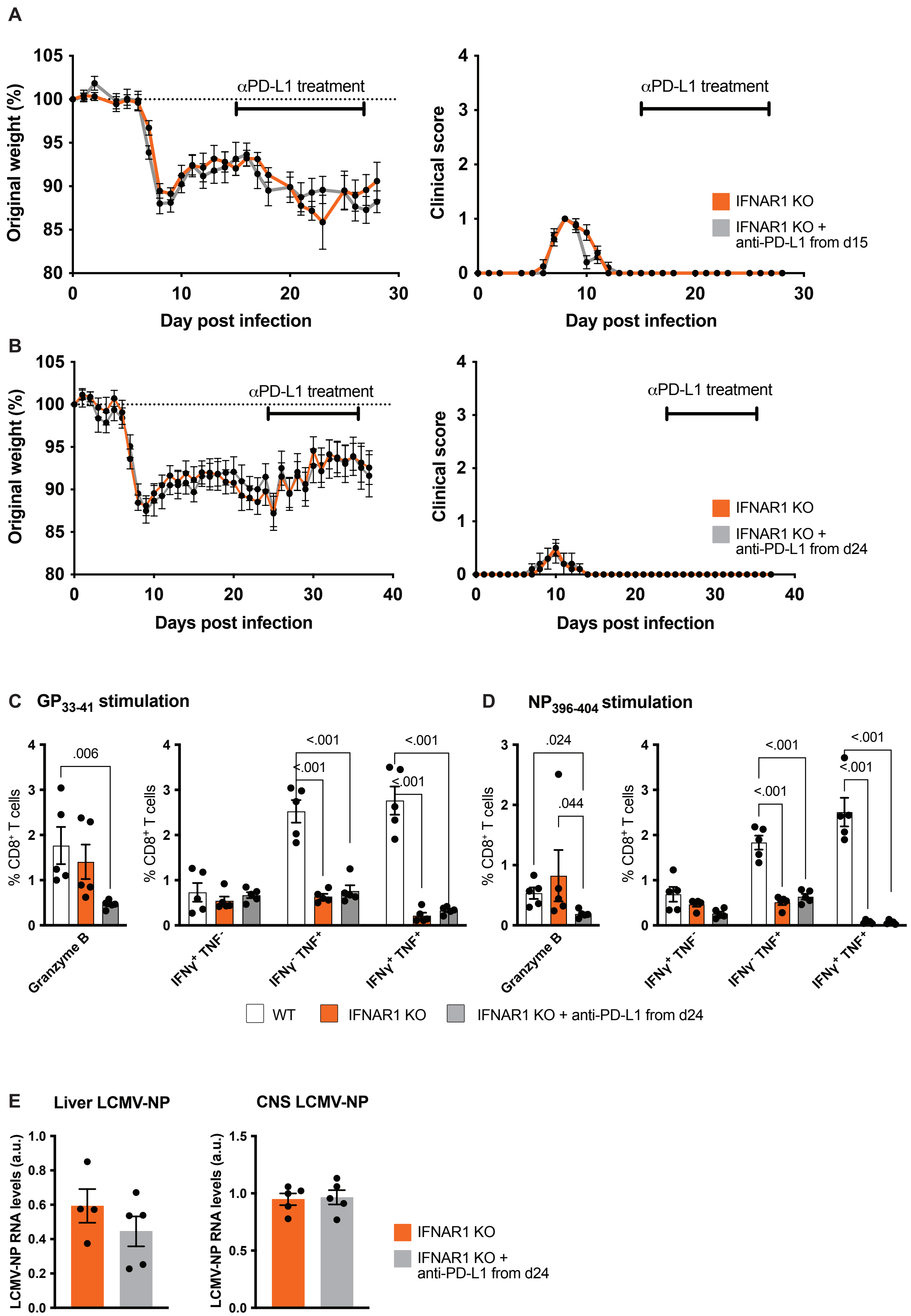

A
Original weight (%)
Day post infection
αPD-L1 treatment
Clinical score
IFNAR1 KO
IFNAR1 KO + anti-PD-L1 from d15
B
Days post infection
IFNAR1 KO + anti-PD-L1 from d24
C GP33-41 stimulation
D NP396-404 stimulation
% CD8+ T cells
Granzyme B
IFNγ+ TNF-
IFNγ- TNF+
IFNγ+ TNF+
.006
<.001
.024
.044
WT
E
Liver LCMV-NP
CNS LCMV-NP
LCMV-NP RNA levels (a.u.)

Figure S3

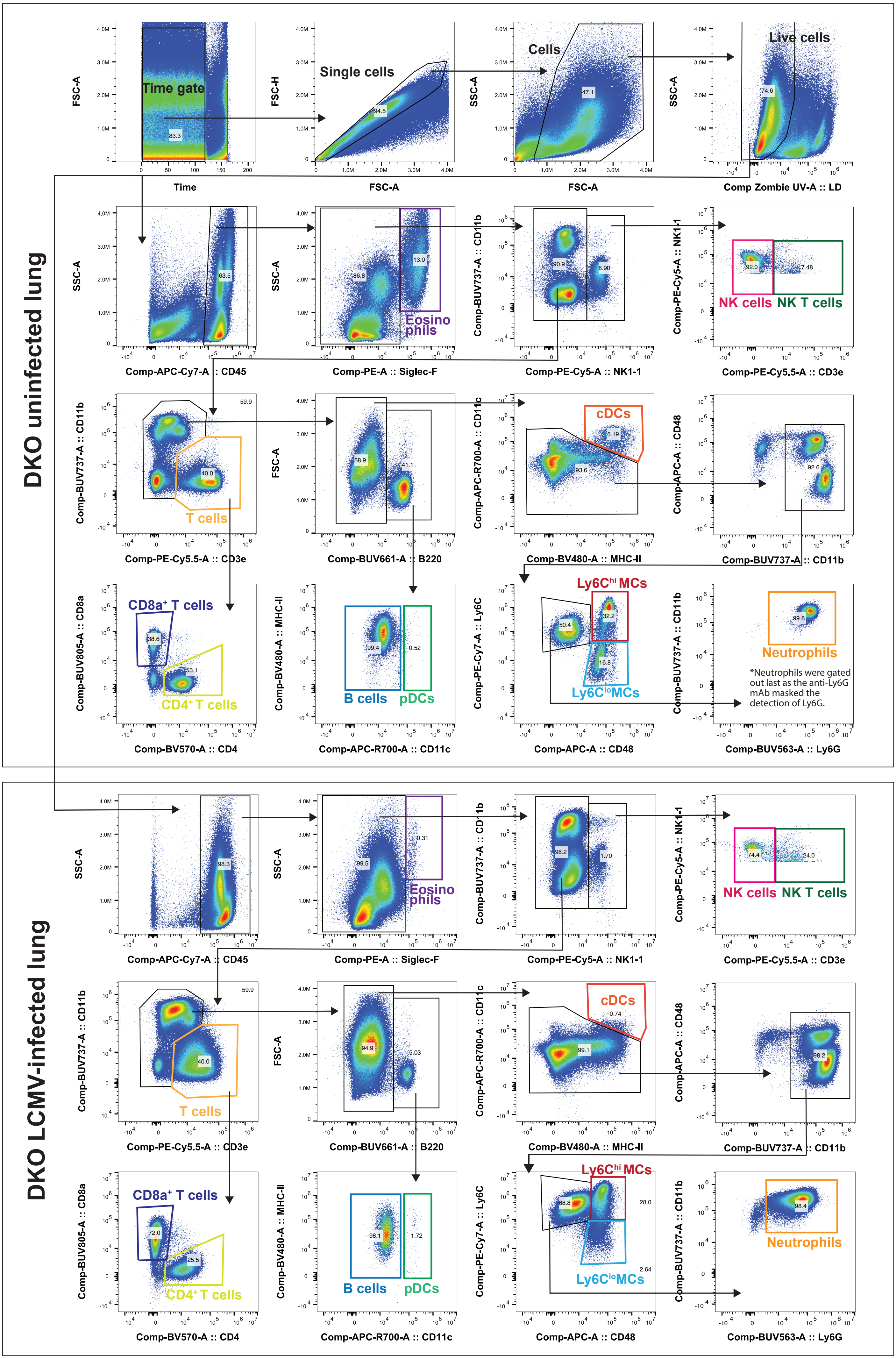

DKO uninfected lung
DKO LCMV-infected lung
Time gate
Single cells
Cells
Live cells
Time
FSC-A
Comp Zombie UV-A :: LD
Eosinophils
NK cells
NK T cells
Comp-APC-Cy7-A :: CD45
Comp-PE-A :: Siglec-F
Comp-PE-Cy5-A :: NK1-1
Comp-PE-Cy5.5-A :: CD3e
T cells
cDCs
Comp-BUV661-A :: B220
Comp-BV480-A :: MHC-II
Comp-BUV737-A :: CD11b
CD8a+ T cells
CD4+ T cells
B cells
pDCs
Ly6Chi MCs
Ly6Clo MCs
Neutrophils
*Neutrophils were gated out last as the anti-Ly6G mAb masked the detection of Ly6G.
Comp-BV570-A :: CD4
Comp-APC-R700-A :: CD11c
Comp-APC-A :: CD48
Comp-BUV563-A :: Ly6G

Figure S4

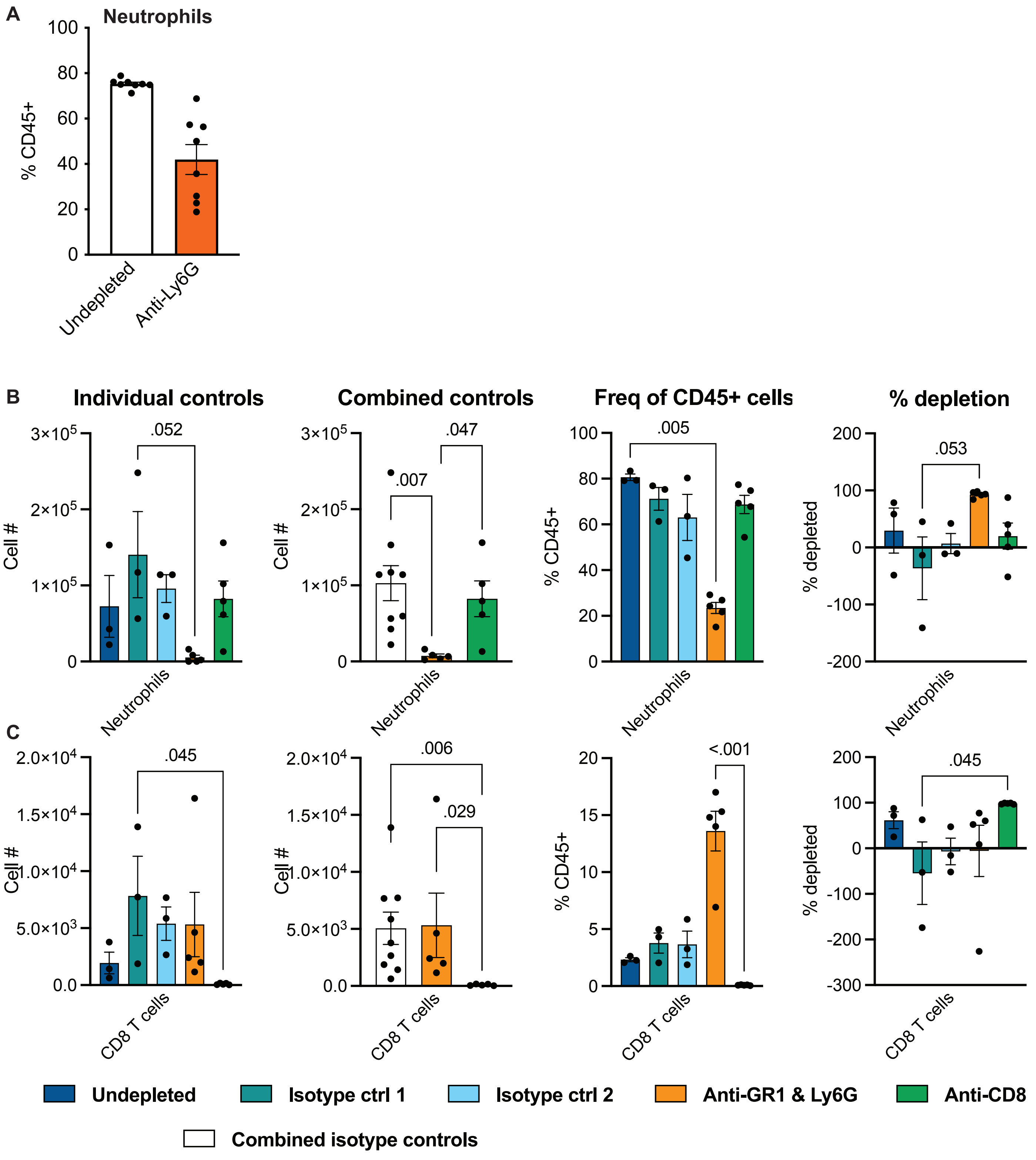

A
Neutrophils
% CD45+
100
80
60
40
20
0
Undepleted
Anti-Ly6G
B
Individual controls
Combined controls
Freq of CD45+ cells
% depletion
Cell #
% CD45+
% depleted
.052
.007
.047
.005
.053
Neutrophils
C
.045
.006
.029
<.001
.045
CD8 T cells
Undepleted
Isotype ctrl 1
Isotype ctrl 2
Anti-GR1 & Ly6G
Anti-CD8
Combined isotype controls

Figure S5

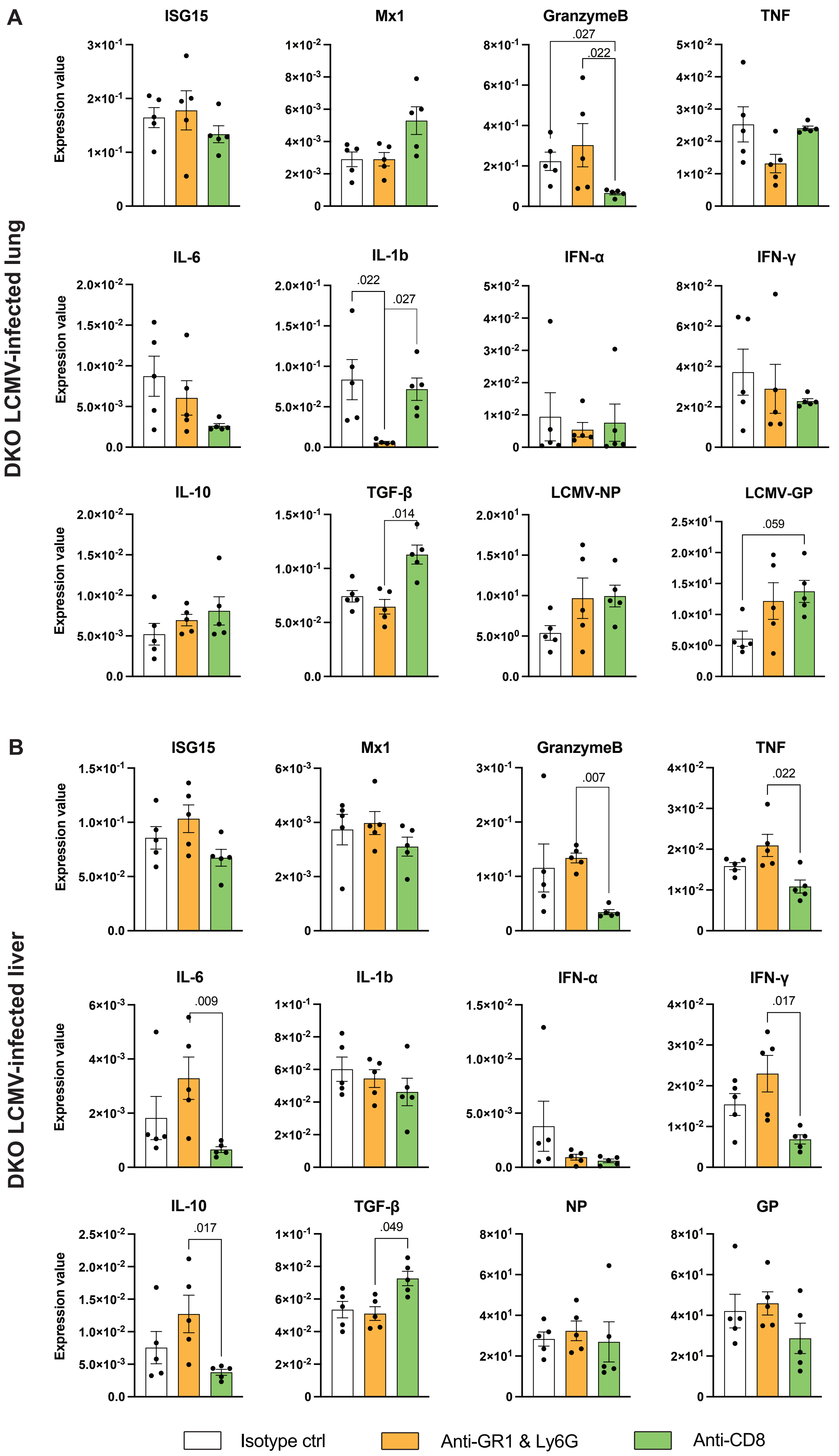
A
B
DKO LCMV-infected lung
DKO LCMV-infected liver
Expression value
ISG15
Mx1
GranzymeB
TNF
IL-6
IL-1b
IFN-α
IFN-γ
IL-10
TGF-β
LCMV-NP
LCMV-GP
NP
GP
.027
.022
.022
.027
.014
.059
.007
.022
.009
.017
.017
.049
Isotype ctrl
Anti-GR1 & Ly6G
Anti-CD8

Supplementary Table 1 Primers used for qPCR

Gene	Sequence
18S	*Forward CACGGCCGGTACAGTGAAAC*<br>*Reverse AGAGGAGCGAGCGACCAA*
Granzyme B	Forward GCTGCTCACTGTGAAGGAAGTAT<br>Reverse GGGATGACTTGCTGGGTCTT
Ifn-γ	Forward GCAAAAGGATGGTGACATGA<br>Reverse TTCGCCTTGCTGTTGCTGA
Ifn-α	Forward GTGACCTTCCTCAGACTCATAAC<br>Reverse GCACTGCCCAACTTGTCTAA
Il-1β	Forward TGGACCTTCCAGGATGAGGACA<br>Reverse GTTCATCTCGGAGCCTGTAGTG
Il-10	Forward AAGGGTTACTTGGGTTGCCA<br>Reverse AAATCGATGACAGCGCCTCAG
Il-6	Forward CAAAGCCAGAGTCCTTCAGA<br>Reverse GATGGTCTTGGTCCTTAGCC
Irf7	Forward GAGACTGGCTATTGGGGGAG<br>Reverse GACCGAAATGCTTCCAGGG
Isg15	Forward GAGCTAGAGCCTGCAGCAAT<br>Reverse TTCTGGGCAATCTGCTTCTT
LCMV-GP	Forward CATTCACCTGGACTTTGTCAGACTC<br>Reverse GCAACTGCTGTGTTCCCGAAAC
LCMV-NP	Forward CAGAAATGTTGATGCTGGACTGC<br>Reverse CAGACCTTGGCTTGCTTTACACAG
Mx1	Forward TCTGAGGAGAGCCAGACGAT<br>Reverse ACTCTGGTCCCCAATGACAG
Pkr	Forward GTTGTTGGGAGGGAGTTGAC<br>Reverse AGAGGCACCGGGTTTTGTAT

Tgf-β	Forward GGAGAGCCCTGGATACCAAC<br>Reverse CAACCCAGGTCCTTCCTAAA
Tnf	Forward GGTGCCTATGTCTCAGCCTCTT<br>Reverse GCCATAGAACTGATGAGAGGGAG

Supplementary Table 2 Antibodies used for flow cytometry

**Target Antigen**	**Conjugate**	**Clone**	**Company and Cat#**
**Spleen panels (ex vivo & stimulation panel)**			
LAG-3	BV421	C9B7W	BioLegend, 125221
CD45	BV510	30-F11	BioLegend, 103138
CD4	BV605<br>BV650	GK1.5<br>RM4-5	BioLegend, 100451<br>BioLegend, 100555
CD179<br>(PD-1)	BV711	29F.1A12	BioLegend, 135231
KLRG1<br>(MAFA)	BV785	2F1/KLRG1	BioLegend, 138429
TNF	FITC	MP6-XT22	BioLegend, 506304
CD44	FITC	IM7	BioLegend, 103006
CD3e	PE/CF594	145-2C11	BD Biosciences, 562332
NK1.1	PE/Cy5	PK136	BioLegend, 108716
IFN-γ	PE/Cy7	XMG1.2	BioLegend, 505826
Dextramer<br>LCMV-GP	APC	NA	Immudex, JA02160
Dextramer<br>LCMV-NP	APC	NA	Immudex, JA02142
Granzyme B	AF647	GB11	BioLegend, 515406
CD8a	APC/Cy7	53-6.7	BioLegend, 100714

Lung panel			
F4/80	BUV395	T45-2342	BD Biosciences, 565614
Ly6G	BUV563	1A8	BD Biosciences, 565707
B220	BUV661	RA3-6B2	BD Biosciences, 565077
CD11b	BUV737	M1/70	BD Biosciences, 564443
CD8a	BUV805	53-6.7	BD Biosciences, 564920
MHC-II	BV480	M5/114.15.2	BD Biosciences, 566088
CD4	BV570	RM4-5	BioLegend, 100542
CD86	BV605	GL1	BD Biosciences, 563055
CD62L	BV650	MEL-14	BD Biosciences, 564108
PD-1	BV711	29F.1A12	BioLegend, 135231
CD69	BV786	H1.2F3	BD Biosciences, 564683
Siglec-F	PE	E50-2440	BD Biosciences, 552126
CD115	AF594	AFS98	BioLegend, 135520
NK1.1	PE/Cy5	PK136	BioLegend, 108716
CD3e	PE-Cy5.5	145-2C11	BD Biosciences, 35-0031-82
Ly6C	PE/Cy7	HK1.4	BioLegend, 128018
CD48	APC	HM48-1	BioLegend, 103411
CD11c	APC R700	N418	BD Biosciences, 565872
CD45	APC/Cy7	30-F11	BD Biosciences, 557659

Blood panel			
CD11b	BUV395	M1/70	BD Biosciences, 563553
CD8a	BUV805	53-6.7	BD Biosciences, 612898
Siglec-F	BV421	E50-2440	BD Biosciences, 562681
CD4	BV570	RM4-5	BioLegend, 100542
Ly6C	BV605	HK1.4	BioLegend, 128036
Ly6G	BV650	1A8	BioLegend, 127641
CD115	PE	AFS98	Biolegend, 135506
CD3e	PE/Cy594	145-2C11	BD Biosciences, 562286
NK1.1	PE/Cy5	PK136	BioLegend, 108716
CD45	AF700	30-F11	BioLegend, 103128
CD48	APC/Cy7	HM48-1	BioLegend, 103432
